# Genomic characterization of polyextremotolerant black yeasts isolated from food and food production environments

**DOI:** 10.3389/ffunb.2022.928622

**Published:** 2022-07-26

**Authors:** Shiyu Cai, Abigail B. Snyder

**Affiliations:** Department of Food Science, Cornell University, Ithaca, NY, United States

**Keywords:** *Aureobasidium*, *Exophiala*, genome duplication, single-gene phylogeny, food mycology

## Abstract

Black yeasts have been isolated from acidic, low water activity, and thermally processed foods as well as from surfaces in food manufacturing plants. The genomic basis for their relative tolerance to food-relevant environmental stresses has not been well defined. In this study, we performed whole genome sequencing (WGS) on seven black yeast strains including *Aureobasidium* (n=5) and *Exophiala* (n=2) which were isolated from food or food production environments. These strains were previously characterized for their tolerance to heat, hyperosmotic pressure, high pressure processing, hypochlorite sanitizers, and ultraviolet light. Based on the WGS data, three of the strains previously identified as *A. pullulans* were reassigned as *A. melanogenum*. Both haploid and diploid *A. melanogenum* strains were identified in this collection. Single-locus phylogenies based on beta tubulin, RNA polymerase II, or translation elongation factor protein sequences were compared to the phylogeny produced through SNP analysis, revealing that duplication of the fungal genome in diploid strains complicates the use of single-locus phylogenetics. There was not a strong association between phylogeny and either environmental source or stress tolerance phenotype, nor were trends in the copy numbers of stress-related genes associated with extremotolerance within this collection. While there were obvious differences between the genera, the heterogenous distribution of stress tolerance phenotypes and genotypes suggests that food-relevant black yeasts may be ubiquitous rather than specialists associated with particular ecological niches. However, further evaluation of additional strains and the potential impact of gene sequence modification is necessary to confirm these findings.

## Introduction

Black yeasts are defined as a functional group, rather than a group defined by phylogeny. For example, black yeasts include the genera *Aureobasidium* and *Exohpiala*, both Ascomycetes, but members of different classes, Dethideomycetes and Chaetothyriomycetes, respectively. These diverse fungi are grouped because of relevant phenotypic attributes, such as their eponymous olivaceous-to-black pigmentation, that contribute to their survival in extreme environments (Cai and Snyder, 2022). Black yeasts are pathogens of animals and plants, relevant to agriculture and food systems, and have been isolated from harsh environments ranging from arctic ice to desert rock surfaces. Due to their polyextremotolerance, black yeast spoilage has been a concern in foods, including refrigerated, frozen, water activity-controlled foods, and shelf-stable products (Snyder et al., 2019b). Even within individual species of black yeasts, there is significant morphological and genetic diversity (Dogen et al., 2013; Balsi et al., 2015; Gostinčar et al., 2019; Cai et al., 2021; Černoša et al., 2021; Cai and Snyder, 2022). Because of the known diversity in environmental source, genetic content, and phenotype, it is possible that only a subset of strains are relevant to specific clinical and agricultural outcomes. Identifying associations between environmental source and black yeast subtype could improve traceback investigations and provide the basis for mitigating product contamination. This underscores the need for rapid and relatively simple methods for subtyping and identification of isolates. Prior research regarding the association between isolation source and the traits for virulence in *A. melanogenum* or halotolerance in *A. pullulans* has been explored (Gostinčar et al., 2019; Černoša et al., 2021). We have posed similar questions in this study but are instead focused on food spoilage outcomes.

Many foods and food production environments are harsh, selecting for only extremotolerant or extremophilic microbiota. For example, many foods and beverages are highly acidic, ranging in pH from ~2 to near neutrality (Breidt et al., 2010; Usaga et al., 2014; Snyder et al., 2019a). Intense thermal processes designed to promote food safety and stability can reach up to 90°C (Biango-Daniels et al., 2019). Some products are preserved through the limitation of water available for microbial growth, quantified through the concept of water activity (a_w_) (Rana et al., 2021). Additionally, microbial persistence on surfaces in the food production environment itself is supported by UV and biocide tolerance (Cai et al., 2020). Nonetheless, black yeasts have been isolated in acidic, low a_w_, and thermally processed foods as well as from surfaces in food manufacturing plants (Snyder et al., 2019b). The genomic basis for tolerance to food-relevant environmental stresses have not been well defined. Individual genes responsible for the proliferation of black yeasts in extreme natural environments are part of a stress-tolerance toolbox that also supports persistence within food processing environments. Though, mediating factors such as individual gene copy number or whole genome duplication are complicating variables relevant to black yeast (Černoša et al., 2021). Understanding the distribution of stress-related genes across isolates may help explain strain-to-strain variation in stress tolerance among black yeasts. Prospectively, this could help the food industry identify problematic strains relevant to their production system. The goal of this study was to 1) evaluate whether there is an association between groups black yeast and food-relevant sources, 2) assess rapid and simple single-locus PCR targets for black yeast subtyping, and 3) determine the distribution of stress-related genes in black yeasts and whether it is correlated with tolerance to food processing strategies.

## Materials and methods

### Fungal cultures

A collection of seven black yeast isolates, including *A. pullulans* (n = 1), *A. melanogenum* (n = 4), *E. dermatitidis* (n = 1), and *E. phaeomuriformis* (n = 1) was assembled. Strains specifically associated with food and food processing environments were preferentially selected. Strains were obtained either from the USDA-ARS Culture Collection NRRL (Northern Regional Research Laboratory) or directly isolated by the authors or their collaborators from commercially produced, spoiled food products (Buehler et al., 2017; Snyder et al., 2019b). Specially, *A. pullulans* 62816 was isolated from plum. *A. melanogenum* Y-12974, Y-9624, FSL-S8-0006, and YB-395 were from seaweed plant, seawater, caramel sauce, and pineapple, respectively. *E. phaeomuriformis* FSL-E2-0572 was isolated from a processed fruit preparation and the isolation source of *E. dermatitidis* YB-734 was unknown. Fungal cultures were stored at −80°C in Potato Dextrose Broth (PDB) (Becton, Dickinson and Co., Sparks, MD) containing 25% (vol/vol) glycerol prior to use. Frozen stock cultures were grown on Potato Dextrose Agar (PDA) (Becton, Dickinson and Co., Sparks, MD) and incubated at 25°C for 28 days. Propagules were harvested by scraped plate collection using 10 ml 0.1% buffered peptone water (Becton, Dickinson and Co., Sparks, MD) per plate. ITS rDNA sequencing was used to confirm the genus of each stock culture received from the USDA NRRL culture collection.

### Purification of DNA

Genomic DNA was extracted from harvested cells of each fungal isolate using standard phenol extraction (Sambrook et al., 1989). Briefly, cell suspensions were centrifuged for 3 min at 4,000 rpm. Supernatant was discarded and the cell pellet was resuspended in 500 µL of DNA Extraction Buffer (Qiagen, Hilden, Germany). This suspension was transferred into a 2 mL screw-top microcentrifuge tube with 600 µL of glass beads and 400 µL of phenol:chloroform:isoamyl alcohol (P:C:I). The mixture was horizontally vortexed for 5 min at high speed followed by centrifugation at 13,000 x *g* for 5 min. Supernatant was then be transferred to a new tube containing 10 µL of 50 mg/mL RNase A and incubated for 60 min at 37°C. After incubation, 300 µL of P:C:I was added to the sample followed by vortexing for 15 s and centrifugation at 13,000 x *g* for 5 min. A mixture of 350 µL of supernatant and 1 mL of 100% chloroform was then transferred to a phase lock tube (Qiagen, Hilden, Germany) and centrifuged at 13,000 x *g* for 5 min. The supernatant containing DNA was washed using 1 mL of ice-cold 100% ethanol and then 1 mL of ice-cold 70% ethanol. The DNA pellet was finally resuspended in 200 µL of sterile ddH_2_O. Quality and concentration was measured using an Invitrogen Qubit Fluorometer (Thermo Fisher Scientific, Liverpool, NY).

### Sequencing, assembly, and annotation

Genomic DNA libraries were constructed using the TruSeq^®^ DNA Nano kit and quantified using the KAPA Biosystem Illumina Library Quantification Kit (KAPABIOSYSTEMS, Wilmington, MA, USA). Libraries were sequenced on an Illumina NextSeq 500 as 2×150 paired-end reads using Illumina version 2.18 (Illumina, San Diego, CA). The raw data was quality checked with FastQC v0.11.8 (Andrews, 2010) and trimmed to remove the adapters using Trimmomatic v0.39 (Bolger et al., 2014). A *de novo* assembly was generated with the clean reads by SPAdes v3.15.2 with the recommended settings *careful* and *k*-mer length 33, 55, 77, 99, and 127 (Bankevich et al., 2012). The quantitative assessment of the genome assembly quality and completeness were evaluated using QUAST v5.0.2 (Gurevich et al., 2013) and BUSCO v5.1.3 (Simão et al., 2015), respectively. All draft genome assemblies were annotated using AUGUSTUS v3.4.0 (Stanke et al., 2008). The short reads for FSL-S8-0006, FSL-E2-0572, NRRL 62816, NRRL Y-9624, NRRL Y-12974, NRRL YB-395, and NRRL YB-734 have been deposited under the “Food Relevant Black Yeast Whole Genome” NCBI BioProject (PRJNA830750) in GenBank under the BioSample accession number of SAMN28416779, SAMN28416780, SAMN28416781, SAMN28416782, SAMN28416783, SAMN28416784, and SAMN28416785.

### Short-read alignment and SNP-based phylogenetic analysis

**Figure 1:**
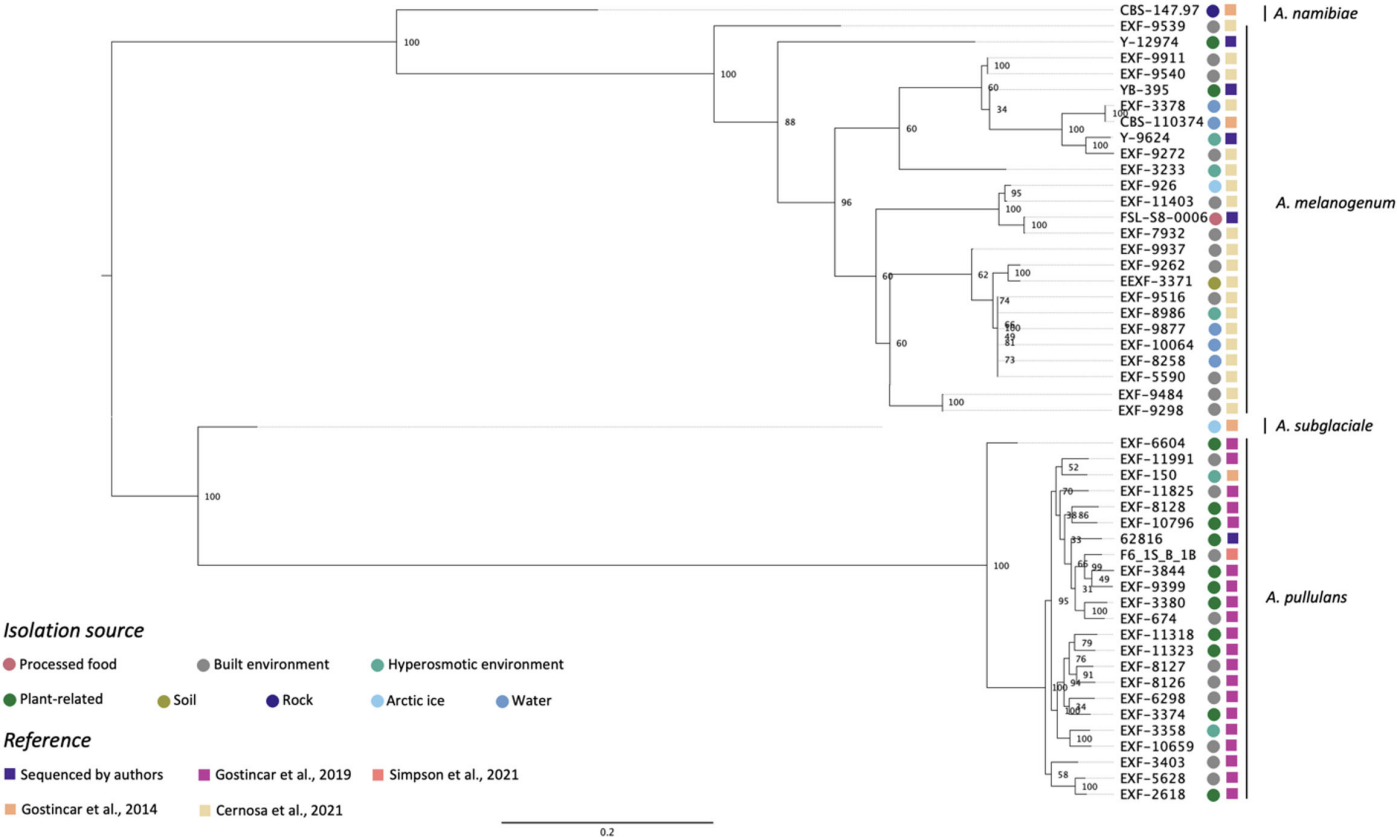
Phylogenetic relationships among *Aureobasidium* strains inferred from SNP alignment. Bootstrap values observed among 1,000 replicates are indicated. The phylogenetic tree was midpoint rooted and the scale bar denotes the number of nucleotide substitutions per site.

**Figure 2:**
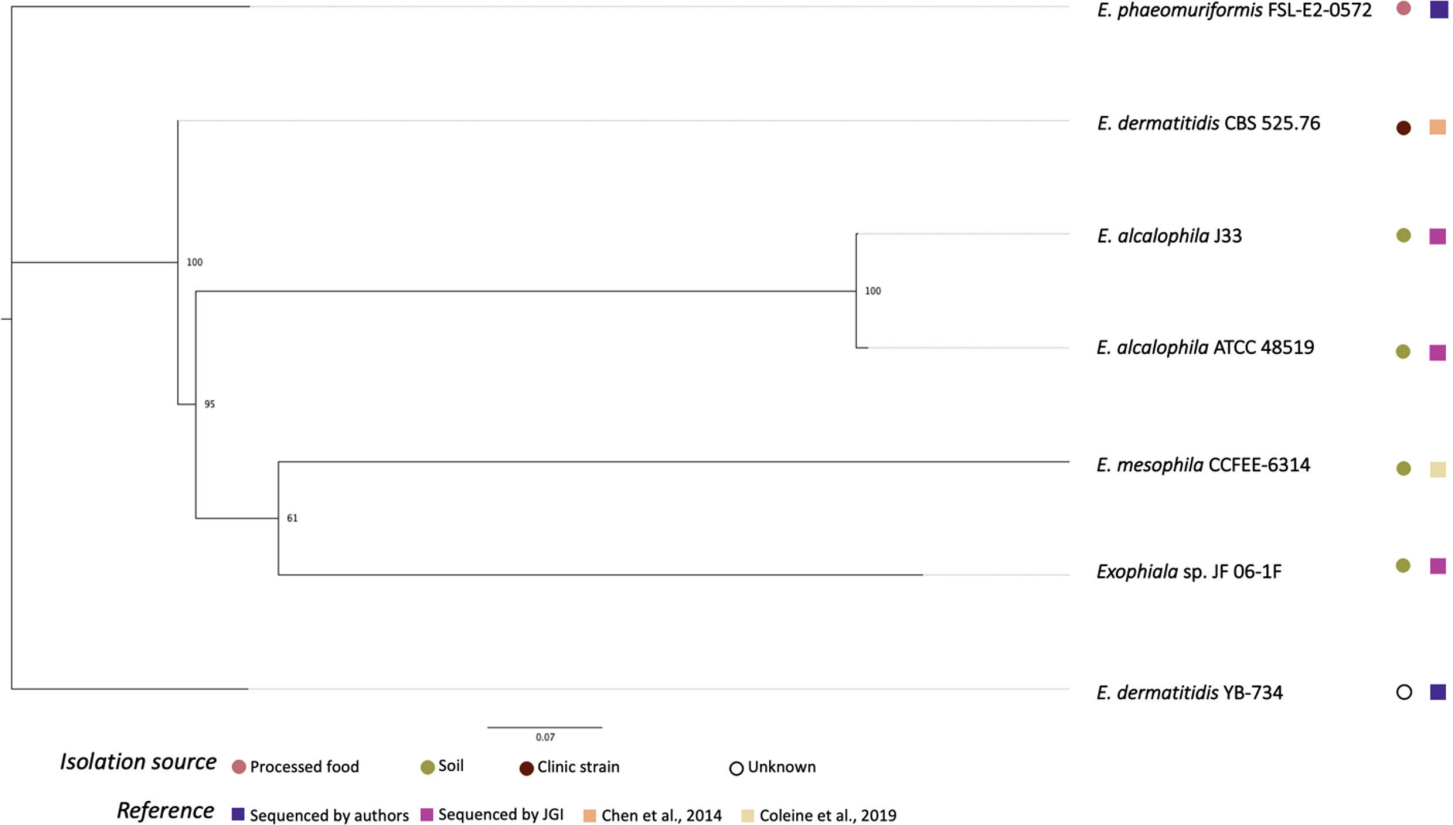
Phylogenetic relationships of *Exophiala* strains inferred from SNP alignment. Bootstrap values observed among 1,000 replicates are indicated. The phylogenetic tree was midpoint rooted and the scale bar denotes the number of nucleotide substitutions per site.

In addition to the seven black yeasts isolates sequenced in this study, WGS data for 48 other black yeast isolates was downloaded from NCBI (Chen et al., 2014; Gostinčar et al., 2014; Nordberg et al., 2014; Coleine et al., 2019; Gostinčar et al., 2019; Černoša et al., 2021; Simpson et al., 2021). Illumina short-reads were mapped onto the genome assembly of *A. pullulans* type strain EXF-150 (Gostinčar et al., 2014) by the pipeline bwa mem (Li, 2013) with the default settings for paired-end data. Subsequently, aligned files were coordinate sorted using sort function from the SAMtools toolkit v0.1.14 (Li et al., 2009), followed by Picard tools v2.26.1 (Broad Institute, 2019) to remove duplicated reads. Single-nucleotide polymorphisms (SNPs) were identified using the mpileup function in BCFtools (with options -a DP,AD –min-MQ 30 –min-BQ 20) (Danecek et al., 2021). The variant calling step was executed using bcftools call function in BCFtools with default settings (Danecek et al., 2021). The SNPs were filtered by applying the bcftools view function with settings of a minor allele frequency less than 0.05, only allowing two alleles, and removing missing genotypes (Danecek et al., 2021). Distance matrixes obtained from the variant calling step produced for **Figures 1**, **2**, and **4** can be found in **Supplementary Materials**, named_under_figure_one_distance_matrix.csv,_figure_two_distance_matrix.csv, and figure_four_distance_matrix.csv, respectively. A Randomized Accelerated Maximum Likelihood (RAxML) tree was constructed based on filtered SNPs with the GTR gamma nucleotide model and 12345 bootstrap replications (Stamatakis, 2014) and visualized using TASSEL 5 v20181005 (Bradbury et al., 2007). The tree was rooted using midpoint rooting by FigTree v1.4.4 (Rambaut and Drummond, 2012). Isolation sources were indicated along the lineages for the categories: processed food, built environment, hyperosmotic environment, plant-related, soil, rock, arctic ice, water, clinical strain, and unknown. More specifically, the “built environment” category contained kitchen appliances, wall surfaces, rubber seals, air, food container surfaces, drains, and dining tables.

### Orthogroup-based phylogenetic analysis

To reconstruct phylogenetic relationships based on secondary identification markers, orthologous gene families were identified from predicted proteome sequences using OrthoFinder v2.5.1 (Emms and Kelly, 2019) under default settings (DIAMOND and -I 5 for tight cluster). Orthologous protein alignments for the beta tubulin, RNA polymerase II, and translation elongation factor sequences in each predicted proteome dataset were obtained from the OrthoFinder v2.5.1 results and concatenated using Geneious Prime v2020.2.3 (Biomatters Ltd., Auckland, New Zealand). A phylogenetic tree was constructed for each enzyme group and were produced with FastTree based on the default species tree method STAG implemented in OrthoFinder. The neighbor-joining consensus trees were reconstructed using the Jukes-Cantor model and 100 bootstrap replications (Stamatakis, 2014). Proteins with unexpected phylogenetic positions or large phylogenetic distances from other proteins in the tree were searched against the non-redundant GenBank protein database (Clark et al., 2016) and their putative function was either confirmed or they were removed from the dataset. The tree was then rooted using a midpoint rooting by FigTree software v1.4.4 (Rambaut and Drummond, 2012).

### Comparative genomic analysis

OrthoVenn2 was used to identify and annotate orthologous gene clusters (Xu et al., 2019). The distribution of shared gene families across selected species was shown in a Venn diagram generated by OrthoVenn2 software. Gene ontology (GO) terms significantly enriched in each strain relative to the other black yeast strains were identified using the GO term enrichment tool on the OrthoVenn2 platform.

### Correlation analysis

The protein sequences of twelve stress tolerance genes as previously reported in Gostincar et al. (Gostinčar et al., 2014) and Gostincar & Gunde-Cimerman (Gostinčar and Gunde-Cimerman, 2018) were downloaded from NCBI for all available *Aureobasidium* sp. and *Exophiala* sp. These sequences were used as queries for a basic local alignment search tool (BLAST) search against the predicted proteomes of the seven black yeast strains using standalone BLAST v2.9.0 (Altschul et al., 1997) with an E-value threshold of 1e-10. The counts of homologues genes per haploid genome were compared among the seven black yeast strains as previously described by Černoša et al., 2021. Spearman correlation analyses were performed in R (Wei et al., 2017). The False Discovery Rate method was used to calculate adjusted *p*-values for the correlation analyses. Visualization of correlation values and their significance were performed with the *corrplot* package in R (Wei et al., 2017).

## Results and discussion

### Species names were reassigned for two *Aureobasidium* strains as a result of the SNP-based phylogenetic analysis

Genome characteristics and assembly quality statistics of the seven food-related black yeast strains were shown in **Table 1**. The assembly size (~50 Mb) of *A. melanogenum* FSL-S8-0006 and *A. melanogenum* NRRL YB-395 was approximately twice the size of the reference haploid genome (~25 Mb). Based on the BUSCO score, these two strains were predicted to be duplicated copies, which suggests that *A. melanogenum* FSL-S8-0006 and *A. melanogenum* NRRL YB-395 are diploid. In contrast, *A. pullulans* NRRL 62816, *A. melanogenum* NRRL Y-12974, and *A. melanogenum* NRRL Y-9624 were predicted single copy according to BUSCO score, suggesting that the strains are haploid. Though, other potential factors can increase genome size in certain strains. The presence of both haploid and persistent diploid *A. melanogenum* strains in nature has been previously reported, although no indication of sexual reproduction has been found within the species (Černoša et al., 2021). The presence of diploid strains is thought to be a consequence of occasional hybridization between relatively heterozygous haploids, while the haploid strains are limited to clonal reproduction (Černoša et al., 2021). Such phenomenon has not been observed in *A. pullulans* and the *A. pullulans* strain sequenced in this study was indeed haploid (Gostinčar et al., 2019). *E. dermatitis* NRRL YB-734 and *E. phaeomuriformis* FSL-E2-0572 have genome sizes of 27.2 Mb and 28 Mb, respectively. The complete BUSCOs of the two *Exophiala* strains were both 98.9% and large shares of the BUSCOs were single-copy.

**Table 1:** Genome characteristics and assembly quality statistics.

Statistic	*Ap* ^a^62816	*Am* ^b^Y-12974	*Am* ^b^Y-9624	*Am* ^b^YB-395	*Am* ^b^FSL-S8-0006	*Ed* ^c^YB-734	*Ep* ^d^FSL-E2-0572
Coverage	162×	210×	114×	84×	84×	83×	75×
Genome assembly size (Mb)	29.4	27.1	26.8	51.7	53.2	27.2	28.0
Number of contigs	392	208	269	687	391	166	286
Contig N50	279,127	753,589	748,340	382,494	398,476	367,521	226,453
GC content (%)	50.27%	50.05%	49.97%	50.27%	50.04%	51.39%	52.83%
Complete BUSCOs	98.80%	99.10%	99.20%	98.90%	99.40%	98.90%	98.90%
Complete and single-copy BUSCOs	98.80%	99.10%	99.10%	3.60%	4.00%	98.90%	98.90%
Complete and duplicated BUSCOs	0.00%	0.00%	0.10%	95.30%	95.40%	0.00%	0.00%
Fragmented BUSCOs	0.40%	0.10%	0.00%	0.40%	0.00%	0.10%	0.10%
Missing BUSCOs	0.80%	0.80%	0.80%	0.70%	0.60%	1.00%	1.00%

a *Aureobasidium pullulans*.

b *Aureobasidium melanogenum*.

c *Exophiala dermatitidis*.

d *Exophiala phaeomuriformis*.

**Figure 1** shows the phylogenetic relationship among five *Aureobasidium* strains sequenced in this study and 45 from previous studies based on whole-genome SNP comparisons. The phylogeny presented four distinct clusters, *A. namibiae*, *A. melanogenum*, *A. subglaciale*, and *A. pullulans*. Out of the five strains sequenced in this study, four clustered with other *A. melanogenum* strains, and one clustered with other *A. pullulans* strains. *A. melanogenum* NRRL Y-12974 and *A. melanogenum* NRRL Y-9624 were originally assigned as *A. pullulans* as they were originally deposited in the NRRL culture collection prior to the emergence of molecular typing tools and modern species concepts. However, based on this analysis, we reassigned these strains as *A. melanogenum.* The four *A. melanogenum* strains all produce black pigmentation after growth on PDA for three days and become more pigmented as the cultures mature (**Figures S1A–C**). By contrast, *A. pullulans* 62816 has pinkish cultures and remains pink throughout maturation (**Figure S1D**). For more than a century, four varieties of *A. pullulans* were described as possessing remarkable variation in morphology and nutritional physiology. These were viz. *A. pullulans* var. *pullulans* (Viala and Boyer, 1891), *A. pullulans* var. *melanogenum* Hermanides-Nijhof (De Hoog and Hermanides-Nijhof, 1977), *A. pullulans* (De Bary) G. Arnaud (De Hoog and Yurlova, 1994), and *A. pullulans* var. *aubasidani* Yurlova (Yurlova and De Hoog, 1997). The species was initially divided into four groups, although the groups do not correlate with morphological differences (Yurlova et al., 1996). There have reportedly been differences in isolation source among the three groups. *A. pullulans* var. *pullulans* was reported to be mostly isolated from sugary or hyperosmotic habitats, such as salterns, fruits, and plant surfaces (Zalar et al., 2008). The var. *melanogenum* has often been isolated from low-nutrient environments, such as moist metal and glass surfaces, showers, fountains, as well as ocean water (Zalar et al., 2008). The var. *subglaciale* is reported to be exclusively from glacial and subglacial ice and sea water (Zalar et al., 2008), while the single isolate *A. pullulans* var. *namibiae* was isolated from marble in Namibia, Africa (Zalar et al., 2008). In 2014, the four varieties were redefined as separate species: *A. pullulans*, *A. melanogenum*, *A. subglaciale* and *A. namibiae* based on genomic analysis and analysis of individual genes genes (Gostinčar et al., 2014). *A. melanogenum* NRRL Y-12974 and *A. melanogenum* NRRL Y-9624 were deposited into the culture collection prior to this taxonomic revision.

**Figure 2** shows the phylogenetic relationships among the *Exophiala* strains sequenced in this study and previous studies. Surprisingly, *E. dermatitis* NRRL YB-734 did not cluster with the clinical isolate *E. dermatitis* CBS 525.76. *E. dermatitis* NRRL YB-734 appeared most likely to be *E. heteromorpha* based on the analysis of ITS rDNA. Historically, the genus *Exophiala* has been characterized by annellidic budding cells (Moussa et al., 2017). Comprising more than 40 species, many *Exohpiala* sp., such as *E. dermatitidis*, *E. oligosperma*, *E. jeanselmei*, *E. lecanii-corni*, *E. phaeomuriformis*, *E. bergeri*, and *E. mesophile*, and *E. xenobiotica* are known to infect humans (Zeng et al., 2007; Kirchhoff et al., 2019). *E. dermatitidis* causes the most systemic infections and is the most frequently isolated *Exophiala* sp., and is, therefore, better studied (Zeng et al., 2007). Due to the diverse morphology of the conidia/conidiophores and sporulation patterns, the taxonomic classification of *E. dermatitidis* has changed frequently. It has previously been classified in the genera *Fonsecaea*, *Hormodendrum*, *Phialophora*, *Rhinogladiella*, *Exophiala*, and *Wangiella* (Hohl et al., 1983). The tropical rain forest has been hypothesized to be the natural reservoir for this fungus, and it has since emerged within the human-dominated environment (Kirchhoff et al., 2019). Even within *E. dermatitidis*, two main genotypes have been detected based on the sequence analysis is *ITS1*. One predominantly consisted of strains from environmental sources, and the other mainly comprised strains from clinical sources. Due to the limited number of *Exophiala* sp. with raw read data readily available, official re-assignment of the species name from *E. dermatitis* NRRL YB-734 requires additional whole genome sequence data for more *Exophiala* strains within several species.

The isolates did not cluster based on isolation source. However, Zalar et al. (2008) stated that *A. pullulans* var. *pullulans* (redefined as *A. pullulans*) are mostly isolated from sugary or hyperosmotic habitats, and the var. *melanogenum* (later redefined as *A. melanogenum*) was often isolated from low-nutrient environments, the isolation sources of the 51 *Aureobasidium* strains in **Figure 1** shows that there are many exceptions to this generalization. For example, three *A. melanogenum* strains were isolated from plant-related environment and processed food. Eleven *A. pullulans* strains were isolated from the built environment or water, where limited nutrients are available. The isolation source of one *A. melanogenum* strain was arctic ice, as is the case of the *A. subglaciale* type strain. Černoša et al. (2021) and Gostinčar et al. (2019) have also reported that no connection exists between geographic habitats and phylogenetic lineages. Those studies each evaluated 50 A*. pullulans* or *A. melanogenum* strains and analyzed phylogenetic lineages based on the whole-genome SNP data. This finding may be attributable to frequent recombination between the widely distributed strains of *A. pullulans* (Gostinčar et al., 2019). In contrast, recombination was not detected within *A. melanogenum* but several hybridization events have been reported between phylogenetically distant haploids (Černoša et al., 2021).

### The duplication of genes adds complexity to single-locus phylogenies

**Figure 3:**
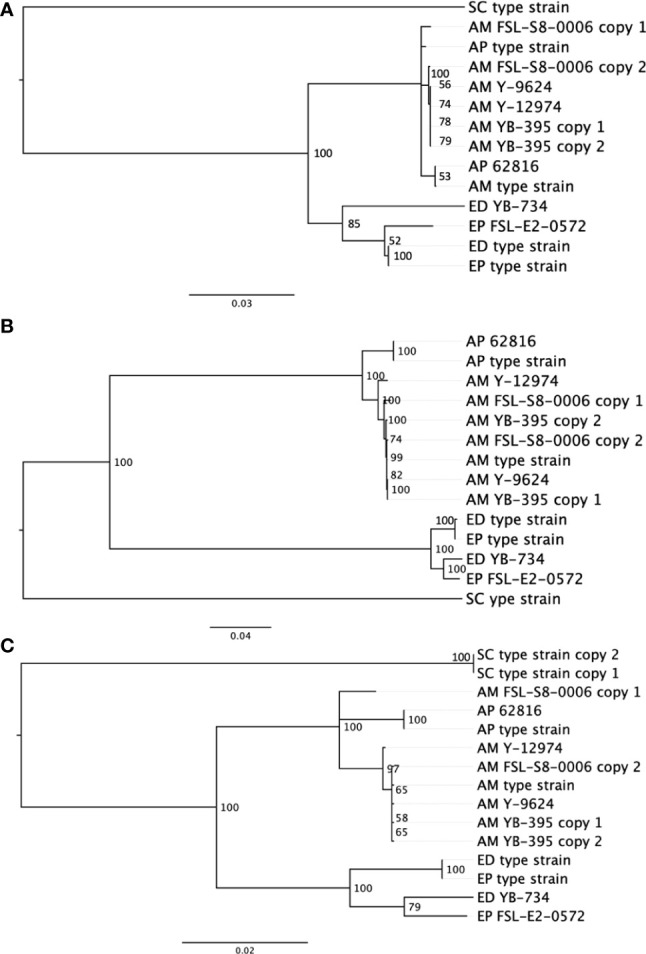
Neighbor-joining phylogenies based on protein sequences for **(A)** beta tubulin, **(B)** RNA polymerase II RPB2, and **(C)** translation elongation factor TEF1 in eight black yeast strains and five other black yeast type strains (AP, *A. pullulans*; AM, *A. melanogenum*; ED, *E. dermatitidis*; EP, *E. phaeomuriformis*; SC, *Saccharomyces cerevisiae*).

In comparison to the SNP-based phylogenetic analysis from short read sequences, an analysis based on single-locus protein sequences was developed to assess an alternative phylogentic method. Activities like traceback investigations are used by the food industry to manage food spoilage. Rapid and simple molecular subtyping methods, such as amplicon sequencing, are more readily adaptable to the industry than are more expensive and computationally demanding approaches such as WGS. We compared the quality of outcomes across three single gene phylogenies (**Figure 3**). The phylogenetic analysis of *BT2*, *RPB2* and *TEF1A* all produced two distinct clusters of proteins, one contained all *Exophiala* strains, and the other contained all *Aureobasidium* strains. Within the *Aureobasidium* clusters, two separate clades were formed with relatively low bootstrap support. *A. melanogenum* FSL-S8-0006 and *A. melanogenum* NRRL YB-395 contained double copies of all three conserved proteins. Each copy of the protein sequence from diploid strains were confirmed by searching against the non-redundant GenBank protein database (Clark et al., 2016). Both copies of the beta tubulin protein sequence from *A. melanogenum* YB-395 clustered closely with one another, while the copies from *A. melanogenum* FSL-S8-0006 were separated into two different clusters. Similarly, the two copies of protein sequence for RNA polymerase II from *A. melanogenum* FSL-S8-0006 were within the same lineage, while the two copies extracted from *A. melanogenum* NRRL YB-395 were divided into two subclusters. The two copies of translation elongation factor TEF1 from *A. melanogenum* NRRL YB-395 were clustered together. Meanwhile, one copy of translation elongation factor TEF1 of *A. melanogenum* FSL-S8-0006 were clustered closer to *A. pulullans* type strain and the other copy clustered closer to *A. melanogenum* type strain. It is assumed that the *A. melanogenum* genome duplication resulted from occasional hybridization between relatively heterozygous haploids (Černoša et al., 2021). Interspecies hybridization may have occurred in *A. melanogenum* FSL-S8-0006.

Due to the low-resolution for *ITS* rDNA as a speciation and subtyping method, other conserved genes have been evaluated and reportedly deliver better resolution in *A. pullulans* compared to *ITS* (Zalar et al., 2008). Coding genes, such as translation elongation factor 1 alpha (*TEF1A*) (Zalar et al., 2008; Manitchotpisit et al., 2009; Yong et al., 2015), β-tubulin (*BT2*) (Zalar et al., 2008; Manitchotpisit et al., 2009; Manitchotpisit et al., 2012; Yong et al., 2015; Rodríguez-Andrade et al., 2019), and the RNA polymerase II second largest subunit (*RPB2*) (Liu and Hall, 2004; Staats et al., 2005; Spatafora et al., 2006; Manitchotpisit et al., 2009; Rodríguez-Andrade et al., 2019) have been used as secondary identification markers for both *Aureobasidium* and *Exophiala* cultures. However, our results suggest some challenges to this approach in black yeast result from duplicated genomes. Although these genes are considered single copy, diploid strains yielded two sequences which, with the exception of beta tubulin sequences, did not consistently cluster together. Diploidy generally complicates phylogenetic reconstruction and would prohibit allelic type analysis. The food industry favors the single-gene phylogeny analysis method compared to SNP-based phylogeny analysis. The single-gene phylogenetic analysis is faster, cheaper, and more conceptually and computationally accessible for industry when compared to SNP-based analysis, and it has already been successfully used in the tracking of bacterial pathogens and spoilage biota (Reichler et al., 2020). Our evidence suggests that the beta tublin sequence may be the most promising single-locus sequence.

### Stress tolerance gene content differed among strains and trends were only observed at the genus level

**Figure 4:**
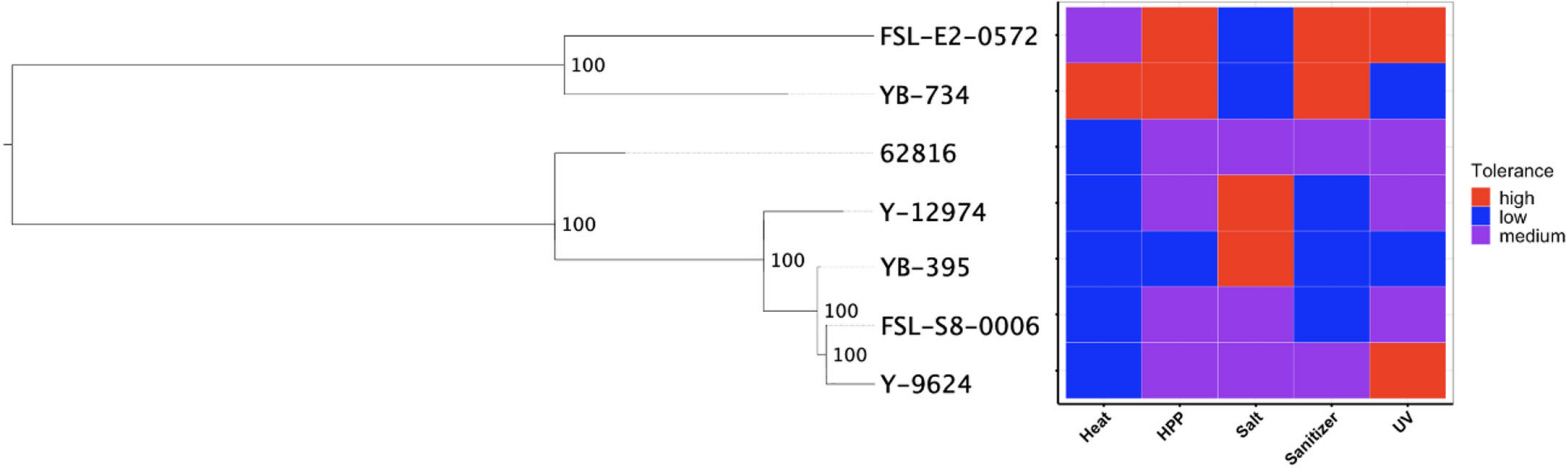
SNP-based phylogeny of black yeast isolated from food or food production environments (left) and their relative stress tolerance to diverse food processing conditions (right).

We hypothesized that clade-specific associations with food-relevant environmental stresses could be identified for black yeast. While certain genus-level differences were identified, the phylogeny for our *Aureobasidium* isolates did not suggest any associations at the species level (**Figure 4**). The relative tolerance of these strains to food processing treatments was previously determined (Cai and Snyder, 2022) and some patterns in phenotype were observed within genera. For example, the two *Exophiala* strains had relatively high thermotolerance, while all *Aureobasidium* strains were relatively susceptible to thermal treatment. Similarly, the two *Exophiala* strains are much more tolerant to high pressure processing (HPP) and hypochlorite sanitizer exposure compared to all *Aureobasidium* strains. By contrast, the *Aureobasidum* strains possessed relatively high halotolerance, while the two *Exphiala* strains were sensitive to increased NaCl concentration. However, the differences between the species of *Aureobasidium, A. pullulans* and *A. melanogenum*, were heterogeneous across different treatment conditions. Consequently, phylogenetic association was not identified with either environmental source (**Figures 1** and **2**) or phenotypic differences (**Figure 4**).

Though further research across a larger collection of food-sourced strains is needed to confirm this finding, this may suggest that the polyextremotolerance and versatility of food-relevant black yeasts is not intraspecific specialization to a specific habitat. This dynamic among black yeast has been recently proposed by Gostinčar et al. (2022) whereby extremotolerant fungi are either generalists (ubiquitous, polyextremotolerant) or specialists (niche specific, targeted extremotolerance), which leverage different predominant strategies of adaption to stress. Our findings may indicate that food-relevant black yeasts are generalists as they are found in many phylogenetic lineages and possess a diversity of stress tolerance phenotypes and genotypes. Food environments exhibit a range of environmental conditions, often cycling between nutrient-abundance and nutrient-scarcity and intermittent hydration. These so-called generalist polyextremotolerant fungi may compete better with mesophilic species, when compared to specialists. However, the identification of generalism is not limited to phylogenetic placement but includes the presence of different functional gene classes.

**Figure 5:**
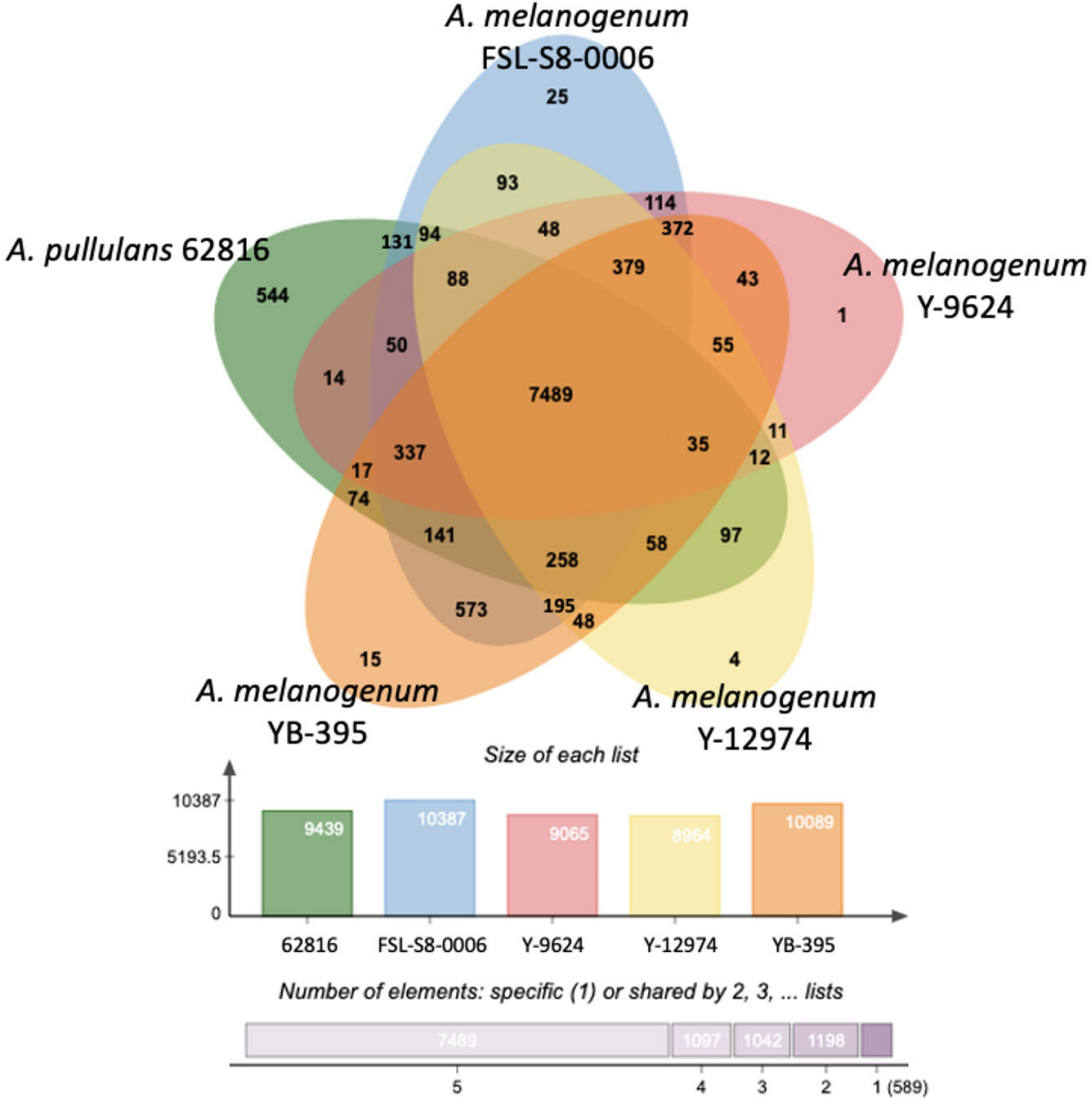
Distribution of unique protein coding genes or pseudogenes in *Aureobasidium* genomes.

To further investigate the variations in the relative functional content of *Aureobasidium*, orthologous gene clusters were compared across species. Despite the differences in ploidy, the five *Aureobasidium* strains had a relatively similar number of protein coding genes or pseudogenes, ranging from 8,964 clusters for *A. melanogenum* NRRL Y-12974 to 10,387 clusters for *A. melanogenum* FSL-S8-0006. The five *Aureobasidium* strains shared 7,489 overlapping orthologous gene clusters representing 72 to 84% of their predicted coding proteins (**Figure 5**). Interestingly, the unique orthologous gene clusters shared between *A. pullulans* 62816 and each *A. melanogenum* strains ranged from 14 to 131, and similarly, the shared unique orthologous gene clusters between each *A. melanogenum* strains ranged from 11 to 114, a similar level of variation in unique orthologous gene clusters both within and between species.

Genes significantly overrepresented within individual strains are listed in **Table 2**. The GO categories for DNA integration (GO: 0015074), metabolic process (GO: 0008152), and L-idonate catabolic process (GO: 0046183) were significantly overrepresented (*p*< 0.05) in *A. pullulans* NRRL 62816 relative to the four *A. melanogenum* strains. Other GO categories, such as translation (GO: 0006412) and ribosome biogenesis (GO: 0042254) were enriched in *A. melanogenum* NRRL YB-395. Consequently, while there is a lack of generalizable difference in gene content among clades, there are notable difference in the functional category of gene content between strains. This functional enrichment suggests that, despite the lack of phylogenetic clustering based on isolation source or phenotype, specialization may still occur through the presence or absence of particular stress-response genes. The possibility of specialization through functional enrichment, as opposed generalization through a shared, versatile set of stress-response genes should be further explored.

**Table 2:** GO terms that are significantly enriched (*p*< 0.05) in each *Aureobasidium* strain relative to other *Aureobasidium* strains.

Strain	GO ID	Category	Enriched GO terms	*p*-value
AP 62816	0015074	biological process	DNA integration	<0.0001
	0008152	biological process	Metabolic process	0.00037
	0046183	biological process	L-idonate catabolic process	0.00040
AM YB395	0042254	biological process	ribosome biogenesis	0.00054
	0006412	biological process	translation	0.00013

### The copy number of stress tolerance genes did not correlate with survival of food processing treatments among the evaluated strains

**Figure 6:**
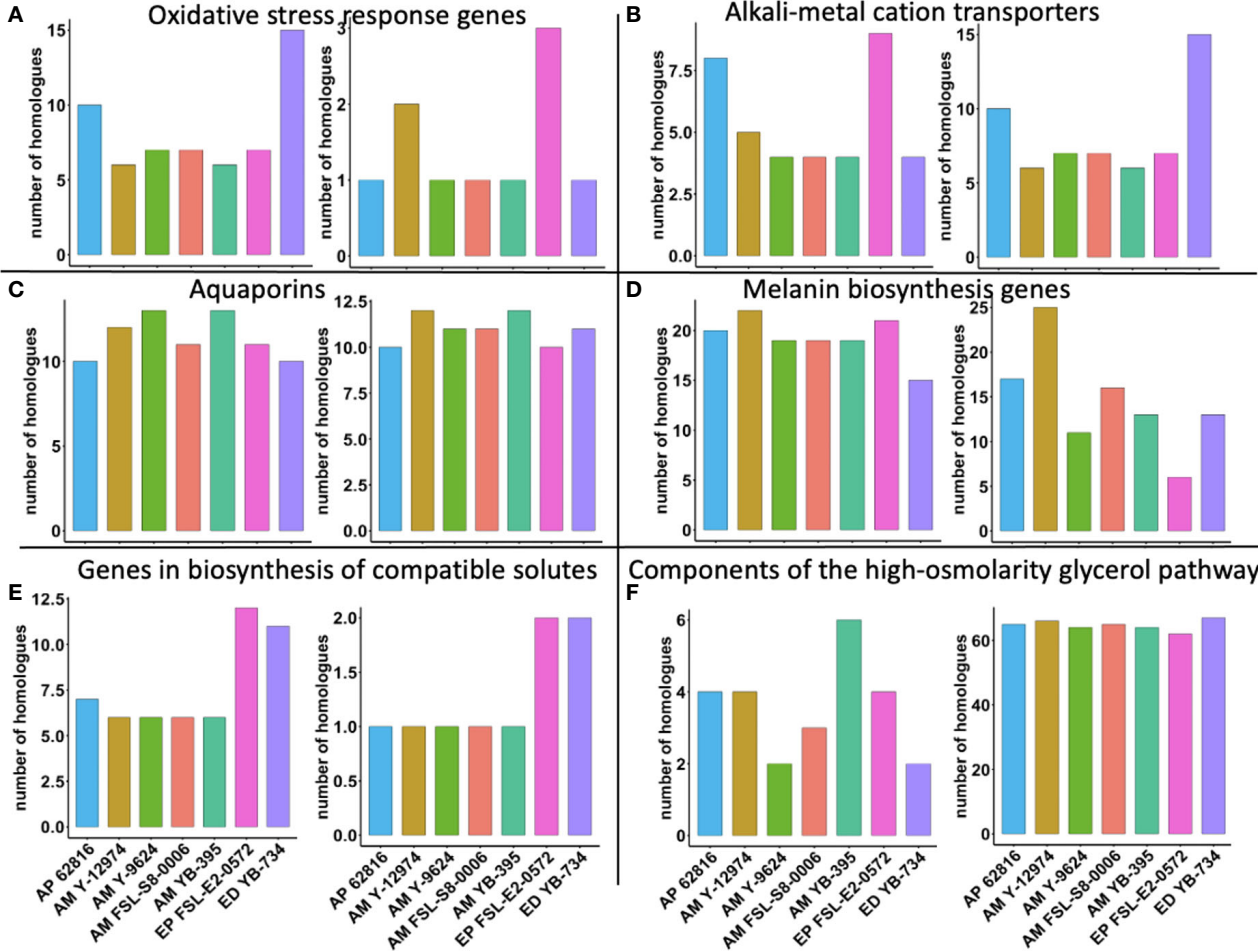
Number of gene homologues in food-relevant black yeast (per haploid genome). **(A)** Oxidative stress response genes: catalase (left) and copper, zinc superoxide dismutase (right); **(B)** Alkali-metal cation transporters: potassium transport protein (left) and cation efflux system proteins (right); **(C)** Aquaporins: aquaglyceroporin (left) and aquaporin (right); **(D)** Melanin biosynthesis genes: laccase (left) and polyketide synthase (right); **(E)** Genes in biosynthesis of compatible solutes: α, α-trehalose-phosphate synthase (left) and NAD-dependent glycerol-3-phosphate dehydrogenase (right); **(F)** Components of the high-osmolarity glycerol pathway: high osmolarity signaling protein (left) and mitogen-activated protein kinase (right).

Finally, we explored whether the copy number per haploid genome of specific stress tolerance genes was associated with food-relevant stress tolerance phenotypes. *E. phaeomuriformis* FSL-E2-0572 had the fewest catalase gene copies, but had the most copper, zinc superoxide dismutase gene copies (**Figure 6**). Oxidative stress response genes have been linked to maximum tolerated salinity among halophilic fungi (Gostinčar and Gunde-Cimerman, 2018). *A. melanogenum* NRRL Y-12974 had the second greatest number of copper, zinc superoxide dismutase gene copies. Meanwhile, *E. phaeomuriformis* FSL-E2-0572 had the most potassium transport protein encoding gene copies and *E. dermatitidis* YB-734 had the most cation efflux system gene copies. The seven strains had similar numbers of laccase gene copies, but *E. phaeomuriformis* FSL-E2-0572 and *A. melanogenum* NRRL Y-9624 had the least polyketide synthase genes copies. The two *Exophiala* strains had doubled numbers of gene copies for α, α-trehalose-phosphate synthase genes and NAD-dependent glycerol-3-phosphate dehydrogenase gene copies compared to *Aureobasidium* strains. In terms of genetic components of the high-osmolarity glycerol pathway, *A. melanogenum* NRRL YB-395 had the highest number of gene copies for high osmolarity signaling protein, while all strains harbors similar numbers of gene copies for mitogen-activated protein kinase.

Several stress tolerance mechanisms, such as melanin biosynthesis, the high osmolarity glycerol pathway, accumulation of compatible solutes, aquaporins, and alkali-metal action transporters have been reported to contribute to black yeasts survival in extreme environments (Bell and Wheeler, 1986; Langfelder et al., 2003; Gostinčar et al., 2014). The variability in the number of gene copy number has been recognized as a source of phenotypic diversity in fungi (Gonçalves et al., 2016; Gostinčar and Gunde-Cimerman, 2018; Steenwyk and Rokas, 2018). For example, an increase in *CUP1* copy number has been associated with stronger copper resistance in *Saccharomyces cerevisiae (* Strope et al., 2015 *)*. The expansion of genes for conidia pigment biosynthesis 1,3,6,8-tetrahydroxynaphthalene reductase gene (*ARP2*) from one to two copies in *Botrytis cinerea* facilitated the synthesis of the sclerotia and conidia melanin (Schumacher, 2016). An evolutionarily recent whole genome duplication and the expansion of genes encoding metal cation transporters was presumed to be beneficial to a halotolerant lifestyle in *Hortaea werneckii (* Lenassi et al., 2013 *)*.

**Figure 7:**
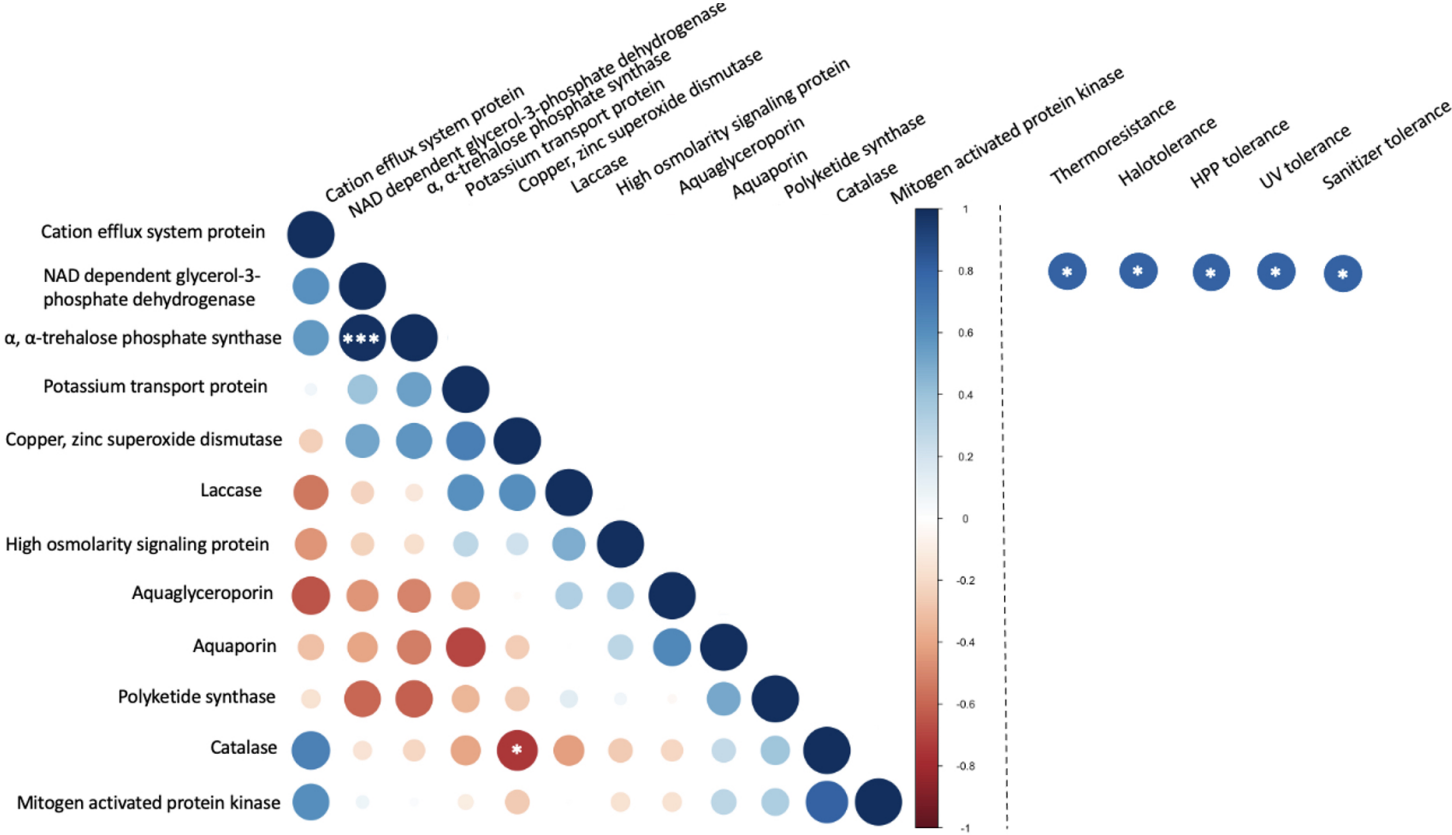
Correlation between the number of stress-related genes in food-relevant black yeast (left) and between gene number and stress tolerance phenotype (right). *p < 0.05; **p < 0.01; ***p < 0.001.

The correlation between copy number and stress tolerance phenotype was further evaluated (**Figure 7**). However, limited instances of correlation between the copy number these selected genes were identified. This included a significant (p< 0.001) positive correlation between the numbers of α, α-trehalose-phosphate synthase genes and NAD-dependent glycerol-3-phosphate dehydrogenase genes. These two enzymes both contribute to pathways in the biosynthesis of compatible solutes. A weaker correlation (p< 0.05) was observed between the copy numbers for catalase and copper, zinc superoxide dismutase. These two enzymes both belong to the oxidative stress response pathway. In the evaluation of associations between the copy number of genes and stress tolerance phenotypes the only detected correlations (p< 0.05) were between NAD-dependent glycerol-3-phosphate dehydrogenase and all five stress tolerance phenotypes but only without correction for multiple comparisons. When the false discovery rate (FDR) correction was applied, none of the correlations between stress tolerance phenotypes and the number of selected genes was considered statistically significant.

## Conclusion

In conclusion, while the findings from this study suggest that food-sourced black yeasts may be ubiquitous rather than isolated to particular ecological niches, additional confirmatory work with a larger collection of isolates is needed. The ecological strategy of generalism may be beneficial in food production environments to reduce fitness trade-off under mesophilic conditions which are intermittently relevant in food production environments. However, only a subset of stress-related genes were evaluated here and genes from other pathways for fungal tolerance and proliferation in extreme environments should also be examined. Notably, the identification of several functional categories of genes overrepresented in certain strains suggests the possibility of specialization through functional enrichment, despite the lack of phylogenetic clustering. Additionally, primary sequence modification to stress-related genes is another avenue for additional exploration.

## Data availability statement

The datasets presented in this study can be found in online repositories. The names of the repository/repositories and accession number(s) can be found below: https://www.ncbi.nlm.nih.gov/genbank/, PRJNA830750.

## Author contributions

SC conducted experiments, collected data, completed the statistical analysis, and drafted the manuscript. SC and AS conceptualized the study, analyzed results, interpreted the findings, and contributed to manuscript revision. AS wrote sections of the manuscript and provided resources and supervision. All authors contributed to the article and approved the submitted version.

## Funding

This work was supported by the National Dairy Council (Rosemont, IL).

## Acknowledgments

The authors thank Qi Sun and Jeff Glaubitz from the Cornell University Institute of Biotechnology (Ithaca, NY) for support in the development of bioinformatic analysis. Fungal cultures were provided by the U.S. Department of Agriculture’s Northern Regional Research Laboratory culture collection and by Professor Martin Wiedmann of Cornell University. We thank both groups for the generous contributions.

## Conflict of interest

The authors declare that the research was conducted in the absence of any commercial or financial relationships that could be construed as a potential conflict of interest.

## Publisher’s note

All claims expressed in this article are solely those of the authors and do not necessarily represent those of their affiliated organizations, or those of the publisher, the editors and the reviewers. Any product that may be evaluated in this article, or claim that may be made by its manufacturer, is not guaranteed or endorsed by the publisher.
