# Supplementary material for: Genomic characterization of polyextremotolerant black yeasts isolated from food and food production environments
Source: Front Fungal Biol. 2022 Jul 26;3:928622. doi: 10.3389/ffunb.2022.928622 (PMC10512282; doi:10.3389/ffunb.2022.928622)
Supplement: Supplementary file 4 [file Image_1.pdf]

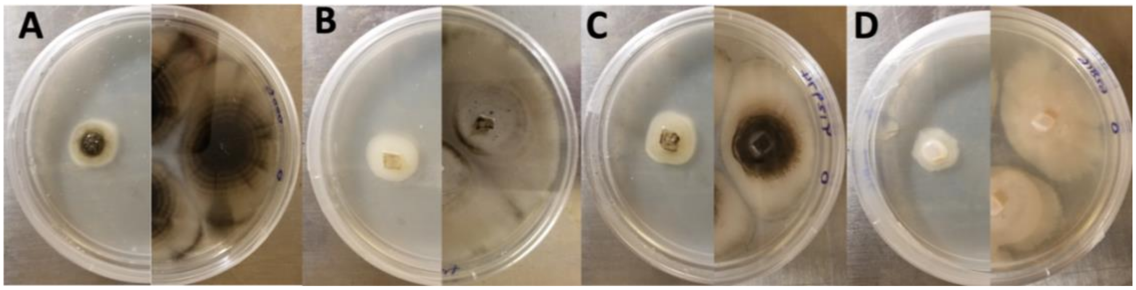

Figure S1. Melanin production of *Aureobasidium* spp. on PDA for three days (left) and 50 days (right). (A) *A. melanogenum* FSL-S8-0006; (B) *A. melanogenum* NRRL Y-9624; (C) *A. melanogenum* NRRL Y-12974; (D) *A. pullulans* 62816.
